# Bone Regeneration Potential of Uncalcined and Unsintered Hydroxyapatite/Poly l-lactide Bioactive/Osteoconductive Sheet Used for Maxillofacial Reconstructive Surgery: An In Vivo Study

**DOI:** 10.3390/ma12182931

**Published:** 2019-09-11

**Authors:** Quang Ngoc Dong, Takahiro Kanno, Yunpeng Bai, Jingjing Sha, Katsumi Hideshima

**Affiliations:** Department of Oral and Maxillofacial Surgery, Shimane University Faculty of Medicine, Izumo, Shimane 693-8501, Japan; dongngocquang1987@gmail.com (Q.N.D.); xyywq@126.com (Y.B.); jsswjbnjw@gmail.com (J.S.); hideg@med.shimane-u.ac.jp (K.H.)

**Keywords:** osteoconductivity, bone defect, bone regeneration, maxillofacial surgery, PLLA, u-HA/PLLA

## Abstract

Uncalcined and unsintered hydroxyapatite/poly l-lactide (u-HA/PLLA) material has osteoconductive characteristics and is available for use as a maxillofacial osteosynthetic reconstruction device. However, its bone regeneration ability in the maxillofacial region has not been fully investigated. This study is the first to assess the bone regenerative potential of osteoconductive u-HA/PLLA material when it is used for repairing maxillofacial bone defects. A total of 21 Sprague-Dawley male rats were divided into three groups—the u-HA/PLLA, PLLA, or sham control groups. A critical size defect of 4 mm was created in the mandible of each rat. Then, the defect was covered with either a u-HA/PLLA or PLLA sheet on the buccal side. The rats in each group were sacrificed at 2, 4, or 8 weeks. The rats’ mandibles were sampled for histological analysis with hematoxylin and eosin staining, histomorphometry, and immunohistochemistry with Runx2 and osteocalcin (OCN) antibody. The amount of newly formed bone in the u-HA/PLLA group was significantly higher than that of the PLLA group. The expression of Runx2 and OCN in the u-HA/PLLA group was also significantly higher. These results demonstrate that the u-HA/PLLA material has excellent bone regenerative ability and confirm its applicability as a reconstructive device in maxillofacial surgery.

## 1. Introduction

The management of maxillofacial bone fractures, reconstructive surgery, and jaw osteotomy usually requires rigid fixation to optimize the healing of bony tissue. Inadequate stabilization of the fracture may lead to non-union or delayed union which is very difficult to treat [1]. To accomplish this task, the role of fixation device material cannot be underestimated. The titanium plates and screws have been the conventional standard fixation measures with superior outcomes owing to its high mechanical strength and osteointegrative nature. However, as a foreign body, titanium hardware is associated with limitations such as stress-shielding, migration, thermal irritability, and infection, so the removal of the device is sometimes necessary [1]. Therefore, resorbable bone plates and screws are theoretically a more desirable option for maxillofacial surgeons and patients if they can fulfil the essential requirements for osteosynthesis [2,3]. Nonetheless, some conventional resorbable materials still have drawbacks, including lower mechanical strength, tissue reaction [4], and early implant mobility [5]. As a result, a more innovative alternative for bone fixation still needs to be developed.

Uncalcined and unsintered hydroxyapatite/poly l-lactide (u-HA/PLLA) is a relatively new material that was introduced in the 1990s and has since been widely used in clinical practice. The material is produced by a forging process to create a composite of u-HA particles and PLLA [6]. This combination brings some favorable characteristics to the material, such as radiopacity, high mechanical strength, biocompatibility, bioresorbability, bone bonding and especially osteoconduction [6]. In fact, u-HA/PLLA possesses a bending strength of 270 MPa, which is greater than the bending strength of human cortical bone, and a modulus of 12 GPa, which is similar to that of cortical bone. Furthermore, the bending strength of u-HA/PLLA can be maintained at 200 MPa until 25 weeks after implantation, which is more than enough for normal bone healing [6]. Regarding osteoconduction, numerous studies using rabbit models have demonstrated the osteoconductive ability and bone bonding features of u-HA/PLLA [7,8,9]. Thus, u-HA/PLLA is used extensively as bone plates and screws for osteosynthesis in different surgical specialties such as orthopedics, plastic surgery, or thoracic surgery [10,11,12,13]. However, these animal experiments only tested the osteoconductive ability of u-HA/PLLA using a rod inserted fully into the knee, such that there was full bone contact. The bone regenerative behavior of u-HA/PLLA in the maxillofacial bone under critical size defect conditions has not been investigated.

From an anatomical and functional point of view, the maxillofacial area is unique, having distinctive components such as teeth and paranasal sinuses that interact with the external environment. In addition, the maxillofacial bone is also involved in mastication and respiration. These features have a substantial impact on reconstruction work. Communitive fracture of the craniomaxillofacial area, fracture of the orbital wall, and surgical movement of the tooth-bearing segment can create bony gaps, such that full contact of the bone with the u-HA/PLLA material is not always achieved, as noted in previous animal investigations. Although osteosynthetic materials made from u-HA/PLLA have been utilized successfully for treating craniomaxillofacial trauma [1,4,14,15,16], and in jaw correction surgery [10,17,18], no animal research using microscopes has been done to assess the osteoconductivity of this novel biomaterial when applied specifically to maxillofacial bone defects. Therefore, we conducted this study to evaluate the bone regenerative potential of a u-HA/PLLA sheet covering a critical-size defect in a rat mandibular angle. The results from this study may help confirm the clinical feasibility and potential outcomes of u-HA/PLLA used in maxillofacial reconstructive surgery and may contribute to new osteosynthetic reconstruction device designs for application in the complex maxillofacial region. 

## 2. Materials and Methods

### 2.1. Materials 

Composite sheets of u-HA/PLLA (Super Fixsorb-MX; Teijin Medical Technologies Co., Ltd., Osaka, Japan) with dimensions of 10 mm length × 10 mm width × 0.1 mm thickness, and PLLA thin film sheets (Teijin Medical Technologies Co., Ltd.), which had the same dimensions, were the two reconstructive materials used in this study. The u-HA/PLLA sheet was a composite made of u-HA and PLLA in which u-HA constituted 40% of the weight while the PLLA sheet consisted of poly l-lactide only. 

### 2.2. Surgical Procedure

A total of 21 Sprague-Dawley (SD) male rats (age = 10 weeks; weight = 250–270 g) were assigned to three groups: (1) u-HA/PLLA group (n = 9), (2) PLLA group (n = 9), and (3) sham control group (n = 3). Each group was divided into three subgroups of 2, 4, or 8 weeks of treatment time. Each subgroup included three rats each from the u-HA/PLLA and PLLA groups and one rat from the sham control group. All rats received general anesthesia via injection with ketamine (90 mg/kg) and xylazine (10 mg/kg) into the intraperitoneum. All operations were performed under standard aseptic conditions. A 1-cm full-thickness longitudinal incision was made through the submandibular skin and the soft tissue was dissected and retracted with forceps to expose the mandibular angle surface. A 4-mm-diameter critical-size defect was created at the mandibular angle using a trephine bur to perforate the mandible from the buccal to the lingual side. Then, the defect was covered buccally as follows: rats in the u-HA/PLLA group received the u-HA/PLLA sheet, whereas rats in the PLLA group received the PLLA sheet. The implants were stabilized in place with hemoclips. In the sham control group, no material was used to cover the defect.

**Figure 1:**
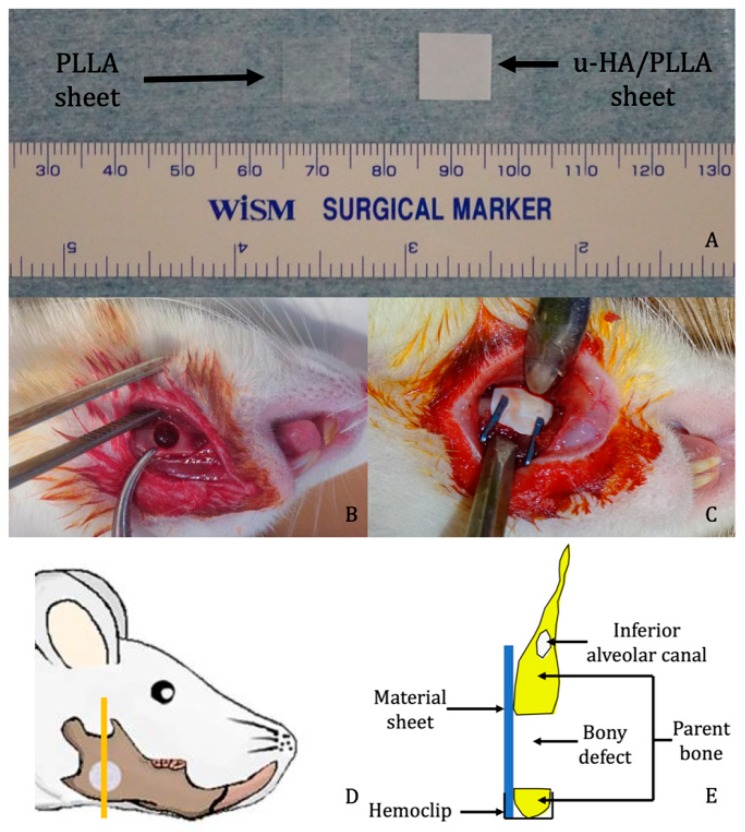
Surgical procedure. (**A**) Examples of unsintered hydroxyapatite/poly l-lactide (u-HA/PLLA) and PLLA sheets. (**B**) Critical size defect created at the mandibular angle. (**C**) Placement of reconstructive material. (**D**) Site from which the sample was taken for analysis (the image was modified from Sha et al. [19]). (**E**) Schematic coronal view of the specimen.

After irrigation with normal saline, the wound was closed in layers. The rats awoke 1–2 h after the operation and exhibited normal behavior and appetite. The healing of all rats was uneventful. All rats were euthanized by administering an overdose of an anesthetic agent at 2, 4 or 8 weeks after the surgery. The mandible was then harvested and soaked in 10% neutral buffered formalin for further analysis (Figure 1).

Surgery and treatment were performed in strict accordance with the Guidelines for Care and Use of Laboratory Animals of Shimane University Faculty of Medicine, Izumo, Japan. The animal protocol was approved by the Animal Ethics Committee of Shimane University (approval references, IZ 26–163 and IZ 28–26).

### 2.3. Tissue Preparation, Hematoxylin and Eosin (HE) Staining, and Immunohistochemistry Staining

#### 2.3.1. Tissue Preparation and HE Staining

The samples from each group at weeks 2, 4, and 8 were decalcified, dehydrated, and embedded in paraffin. The specimens were sectioned along the coronal plane such that each final section contained the defect, the upper and lower host bone, and the reconstructive sheet (Figure 1E). The sections were stained with HE for histological evaluation and histomorphometry.

#### 2.3.2. Immunohistochemistry Staining with Runx2 and Osteocalcin (OCN)

The paraffin-embedded tissue specimens were cut into 4-mm sections. The sections were deparaffinized with xylene and rehydrated with ethanol. Enzymatic antigen retrieval was carried out using proteinase K (0.4 mg/mL). Then, 3% hydrogen peroxide solution was used to block endogenous peroxidase activity. The sections were then incubated with rabbit polyclonal anti-Runx2 (1:1000; ab23981; Abcam, Cambridge, UK) or mouse monoclonal anti-OCN (1:1000; clone: OCG3; ab13420; Abcam) overnight at 4 °C. After three washes with phosphate-buffered saline, the sections were incubated with Histofine Simple Stain MAX PO (MULTI) (414191; Nichirei Biosciences Inc., Tokyo, Japan) for 30 min at room temperature. Finally, the sections were incubated with diaminobenzidine (DAB) for 10 min and counterstained with hematoxylin for 2 min. All immunohistochemistry procedures were conducted by Sept. Sapie Co., Ltd. (Tokyo, Japan). The stained slices were observed using a BX43 light microscope (Olympus Corp., Tokyo, Japan). 

### 2.4. Histomorphometric Evaluation

**Figure 2:**
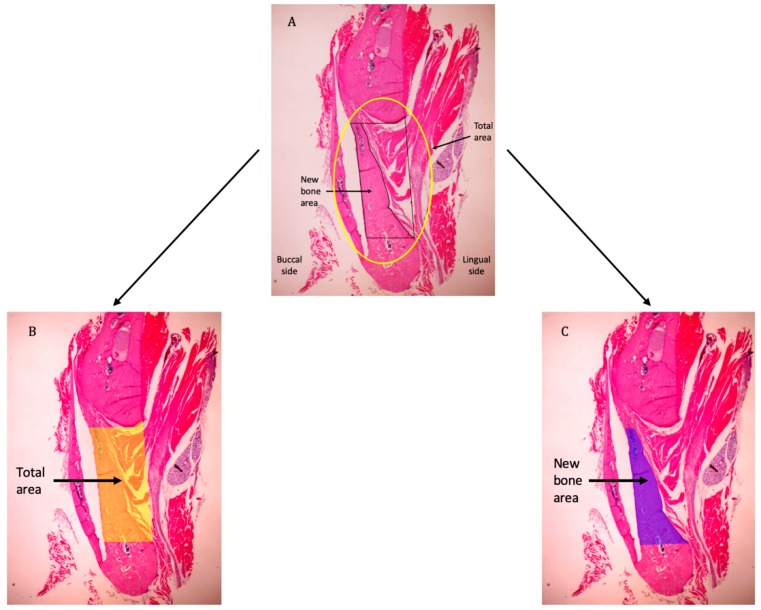
Histomorphometric assessment. The total area consists of the space bordered by the upper and lower bone edges, the lingual side of the reconstructive material, and the line connecting the lingual edges of the bone stumps. (**A**–**C**) Example images showing selection of the total area and new bone area.

Histomorphometry was performed to quantify the amount of new bone formed within the defect region. One picture was taken for each slice using a high-resolution camera (Microscope Digital Camera DP21; Olympus Corp.) mounted on the microscope at 4× magnification. Each picture included the whole defect, which was bordered by the upper and lower bony margins of the defect, the sheet covering the defect, and the lingual periosteum. All pictures were saved to a computer in TIFF format. They were analyzed using an ImageJ plugin distributed as a part of the Fiji project (https://imagej.net/Fiji) [19,20]. The analyzing process was similar to that in the 2018 study by Gavazzoni, Filho, and Hernanes [21]: The total area as mentioned above (i.e., the whole defect) and the new bone area were selected using the selection tool in Fiji. Then, these selections were stored using the ROI manager tool. After that, the total areas were measured, and the percentage of new bone was calculated (Figure 2). This analysis was performed on all pictures taken from the rats sacrificed at weeks 4 and 8, because bone formation in both groups at week 2 was minimal or non-existent.

### 2.5. Immunohistochemical Assessment

The expression of Runx2 was quantified using a labeling index. Three photographs were taken at each of the following regions: the upper and lower bone margins, the defect region adjacent to the reconstruction sheet, and the center of the defect. Each picture was acquired under at 40× magnification; 12 pictures were taken of each specimen. All pictures were stored in TIFF format and analyzed using Fiji software. Specifically, each picture was loaded to Fiji and the Cell Counter plug-in was used to count all positive and negative cells in the photograph area. Then, the percentage of positive cells was calculated. The average percentage of all pictures from one specimen was the labeling index for that particular specimen. 

OCN is deposited mainly in the extracellular matrix [22] of bony tissue. The expression of OCN in the new bone area was measured using the digital H-score following the methods of Fuhrich, Lesley, and Savaris (2013) and Nguyen et al. (2013) [23,24]. This method quantifies the intensity of the DAB chromogen stain using the intensity function in the Fiji software and is based on the fact that a higher DAB intensity indicates a higher concentration of antigen. Numerically, a high-intensity DAB signal, which is darker in color, will have a lower value on a scale from 0 to 255. Therefore, the two aforementioned studies suggest using the digital H-score, i.e., reciprocal intensity, to indicate the level of antigen presence. 

**Figure 3:**
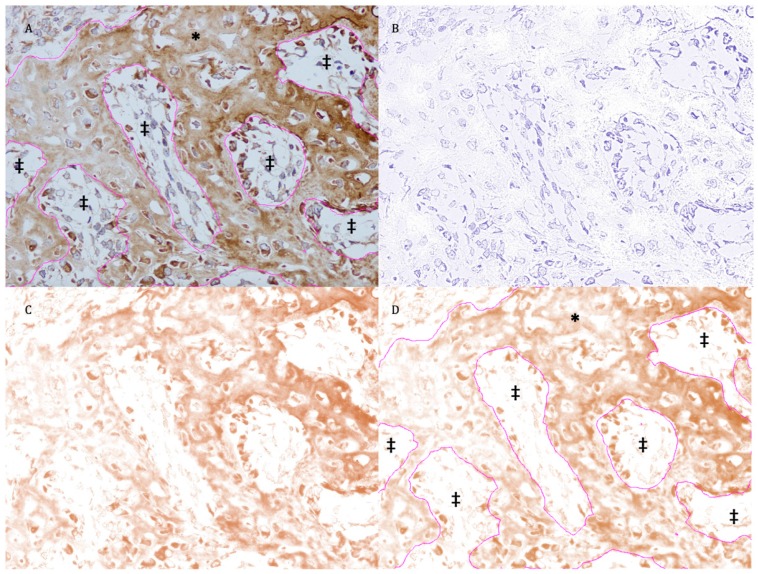
Example new bone area selected as the region of interest. (**A**) Original image with overlay of the selected region of interest. * Selected area; ‡ unselected area. (**B**) Hematoxylin-stained image separated from the original photograph. (**C**) Diaminobenzidine (DAB)-stained image separated from the original photograph. (**D**) Superimposition of the region of interest onto the DAB-stained image. * Selected area; ‡ unselected area.

To calculate the digital H-score, an empty area was first selected and its RGB values were checked. If the values were not close to 255, the “Subtract Background” command (Process/Subtract Background) should be used to fix the uneven background. Then, the region of interest (the new bone area) was selected using various selection tools (Figure 3A). The selection was saved to the ROI manager. Next, the “Colour Deconvolution” function (Image/Colour Deconvolution) with an H DAB vector was used to separate the image into three panels representing the hematoxylin-stained image (Figure 3B), the image stained with DAB only (Figure 3C), and the background. The DAB image was selected, and the previously selected region of interest was superimposed onto the DAB image (Figure 3D). After that, the “Measure” function in the ROI manager menu was used to quantify DAB intensity (i), which ranged from 0 (black) to 255 (white) (Figure 3D). The digital H-score (f), or the reciprocal intensity, of all new bone areas in each specimen was then calculated using the formula f = 255 − I, as mentioned by Nguyen et al. (2013) [24].

### 2.6. Statistical Analyses

Statistical analyses were performed using SPSS software for Mac OS (version 20.0; IBM Corporation, Armonk, NY, USA). The Mann-Whitney U test was used to compare the percentage of new bone (histomorphometry), labeling index (Runx2), and digital H-score (OCN) between the u-HA/PLLA and PLLA groups at different time points. An intra-group comparison was also carried out. A p-value of less than or equal to 0.05 was considered to indicate significance. 

## 3. Results

### 3.1. Histological Evaluation

**Figure 4:**
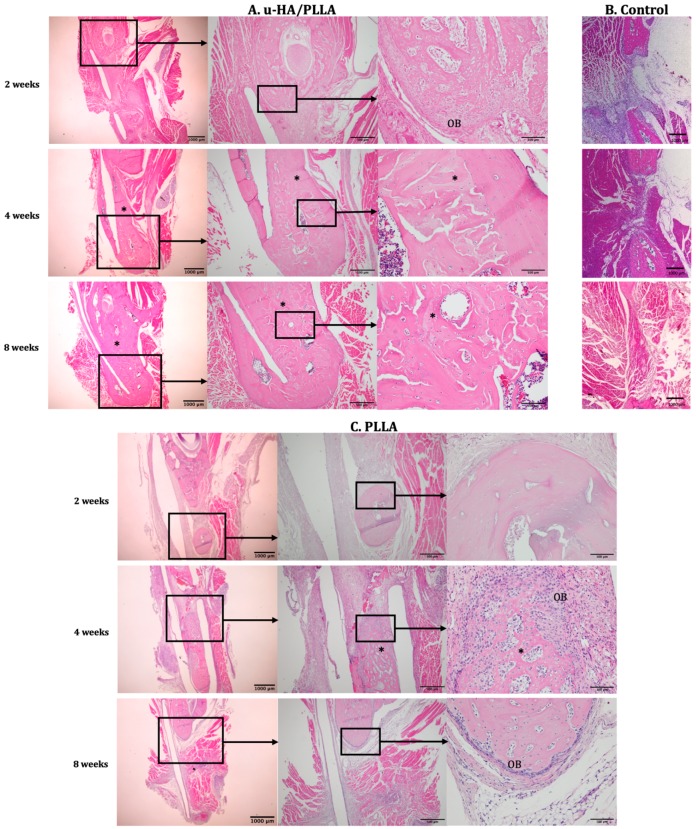
Hematoxylin and eosin-stained sections from the u-HA/PLLA, PLLA, and sham control groups at weeks 2, 4, and 8. The photographs in each subgroup were taken at 1.25×, 4×, and 20× magnification (from left to right). Photographs of the sham control subjects were taken at 1.25× magnification. (**A**) The u-HA/PLLA group, (**B**) the sham control group, and (**C**) the PLLA group. The u-HA/PLLA group exhibited a significant amount of new bone formation at weeks 4 and 8, whereas the PLLA group began to exhibit limited new bone formation at week 4. The control rats exhibited no new bone formation, limited connective tissue, and migration of muscle cells from both side at all time points. * New bone; OB, osteoblast.

At week 2, the u-HA/PLLA-treated rats exhibited a small amount of new bone formation at the center of the defect. Trabecular bone and newly formed blood vessels were present at the defect-margin of the parent bone. The area below the periosteum of the bone stumps had a high concentration of osteoblasts in the active state. The u-HA/PLLA sheet was surrounded by connective tissue. By contrast, in the PLLA-treated rats, lamellar bone with a few osteocytes were present at the bone margin, with some flat osteoblasts at the periphery. The defect was filled with mainly connective tissue. No new bone was observed (Figure 4C). Observations of the sham control subjects were similar to those of the PLLA group. The bone stumps had lamellar bone features with resting osteoblasts located inside the periosteum. Limited connective tissue was found at the defect, which was taken over by muscle on both sides. No new bone was observed.

At week 4, few osteocytes were observed at the bone margins of most subjects in the u-HA/PLLA group, with a thin layer of flat osteoblasts at the periphery. Nevertheless, large numbers of osteoblasts were found at the margin in some specimens, similar to week 2 results. Some specimens had large amounts of new bone formation at the center of the defect with a lamellar bone pattern. The new bone was indistinguishable from the parent bone and it was deposited directly on both sides of the u-HA/PLLA sheet (Figure 4A). The PLLA group began to exhibit features similar to those of the u-HA/PLLA group at week 2 but to a lesser extent. Some new bone formation was seen inside the defect attached to the PLLA sheet. The new bone was mostly immature with a low level of mineralization and was surrounded by a large number of osteoblasts. The sham control subjects exhibited similar features at week 4 to those observed at week 2. No new bone could be seen at the center of the defect because of the herniation of the masseter and the medial pterygoid muscle filled the defect space (Figure 4B).

At week 8, a large amount of new bone could be seen in the u-HA/PLLA-treated specimens. The bone had a lamellar pattern and it attached to both sides of the material and even surrounded the bottom end of the sheet. The new bone completely filled the defect in some specimens and the appearance of the new bone could not be differentiated from the parent bone (Figure 4A). In some specimens, new trabecular bone with a low level of mineralization and numerous osteoblasts was also observed at the buccal side of the material sheet, which was outside the defect. The new bone was continuous with the defect margin of the parent bone and had mature features. However, in some specimens, trabecular bone and osteoblasts with large nuclei were observed inside the periosteum. The PLLA group, on the other hand, exhibited characteristics similar to the u-HA/PLLA group at week 2 with regard to the defect margin of the parent bone. Minimal new bone was detected in the defect area. In some subjects, the defect was occupied by the medial pterygoid muscle from the lingual side (Figure 4C). No new bone could be seen in the sham control subjects. For these rats, defects were completely filled by muscles on both sides. No connective tissue was observed in these subjects.

### 3.2. Histomorphometry

**Figure 5:**
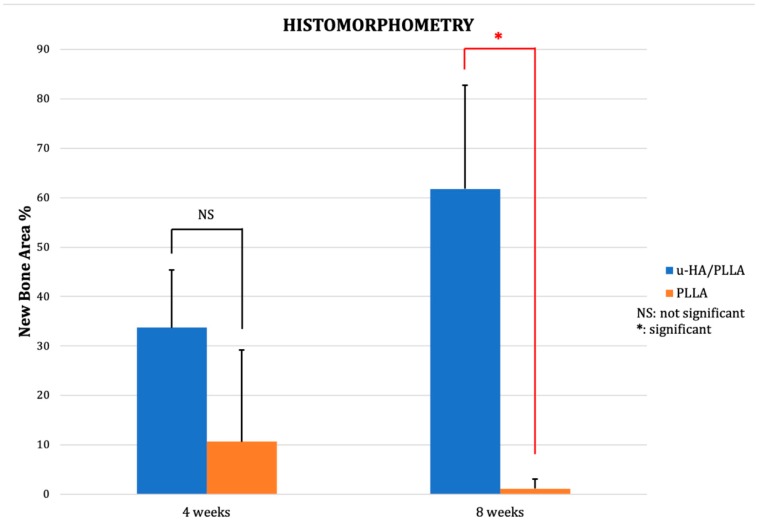
Percentage of new bone area in the u-HA/PLLA and PLLA groups at weeks 4 and 8. * *p* < 0.05.

At week 2, there was either minimal (u-HA/PLLA group) or no (PLLA group) new bone formation, thus the histomorphometric assessment was carried out only with rats sacrificed at weeks 4 and 8. At week 4, the average new bone formation observed in the u-HA/PLLA group (33.74%) was higher than that of the PLLA group (10.69%). However, this difference was not statistically significant. At week 8, the average percentage of new bone area over total area for the u-HA/PLLA group rose to 61.77%, which was significantly higher than that of the PLLA group (1.14%; *P* = 0.012) (Figure 5).

### 3.3. Immunohistochemistry

#### 3.3.1. Runx2

**Figure 6:**
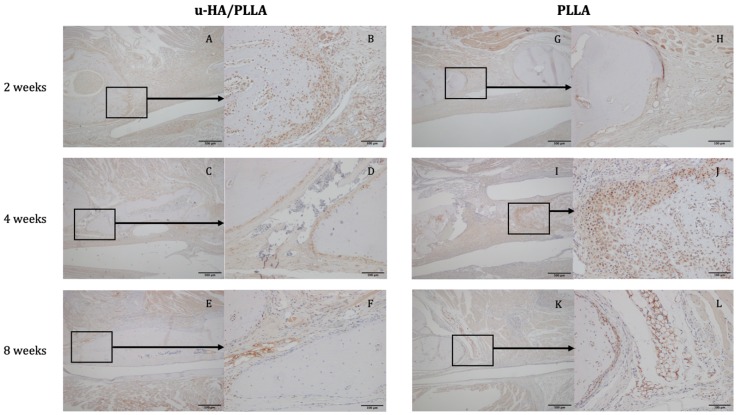
Expression of Runx2 in the u-HA/PLLA and PLLA groups. (**A**–**F**): In the u-HA/PLLA group, Runx2 expression decreased significantly from weeks 2 to 8. (**G**–**L**): Runx2 expression in the PLLA group was generally lower, except in specimens with new bone. (**A**,**C**,**E**): Images of the u-HA/PLLA-treated samples taken at 4× magnification at weeks 2, 4, and 8, respectively. (**G**,**I**,**K**): Images of PLLA-treated samples taken at 4× magnification at weeks 2, 4, and 8, respectively. (**B**,**D**,**F**,**H**,**J**,**L**): Images of the boxed regions in (**A**,**C**,**E**,**G**,**I**,**K**), respectively, at 20× magnification.

**Figure 7:**
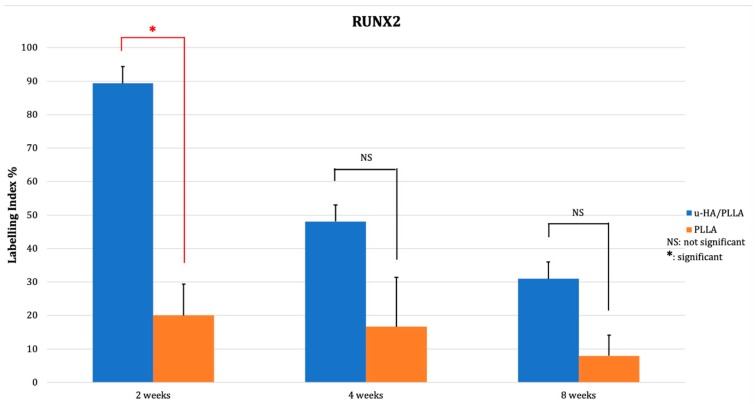
The results of immunohistochemical staining with anti-Runx2 antibody. * *p* < 0.05.

The expression of Runx2 in the u-HA/PLLA group was 89.34% at week 2. The positive cells were concentrated in the parent bone at the upper and lower aspects of the defect where the osteoblasts were located (Figure 6A,B). Runx2 was also expressed at the center of the defect and near the u-HA/PLLA sheet. The labeling index decreased to 48.08% at week 4 and 30.96% at week 8. At weeks 4 and 8, Runx2 was mainly expressed at the periphery of the parent and new bone in the u-HA/PLLA group (Figure 6C,D and Figure 6E,F, respectively). Conversely, Runx2 was weakly expressed in the PLLA group, with positive cells only present at the periphery of the parent bone at week 2 (Figure 6G,H). At week 4, one specimen exhibited higher Runx2 expression associated with the formation of new bone (Figure 6I,J). However, the other specimens showed low expression. A low level of expression could also be observed in PLLA group at week 8 (Figure 6K,L). The labeling index of Runx2 in the PLLA group declined slightly from 20.03% at week 2 to 16.69% at week 4 and to 6.18% at week 8. The difference between the two groups at week 2 was statistically significant (*p* < 0.05). The labeling index of the u-HA/PLLA group at week 2 was also significantly higher than at week 8 (*p* < 0.05) (Figure 7). 

#### 3.3.2. OCN

**Figure 8:**
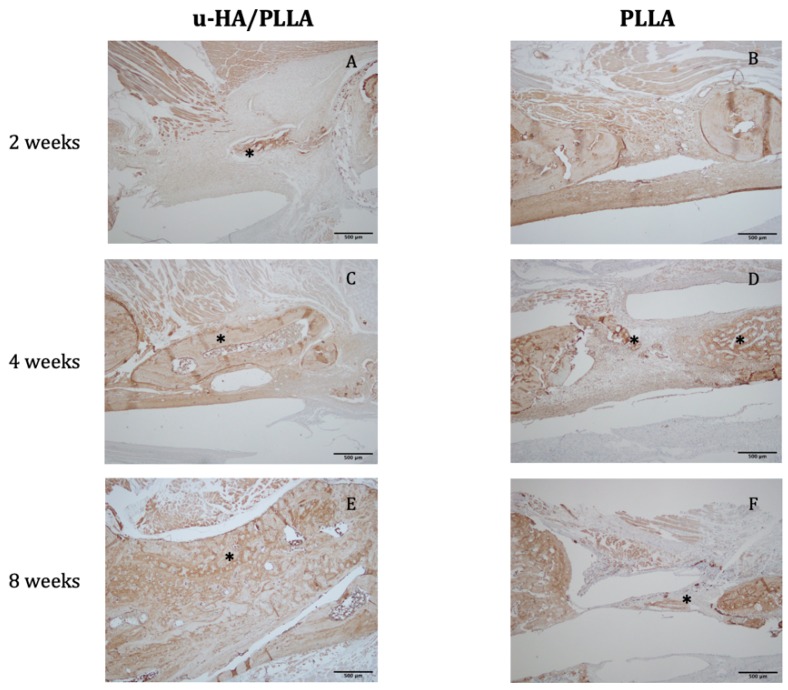
Expression of osteocalcin (OCN) in the (**A**,**C**,**E**) u-HA/PLLA and (**B**,**D**,**F**) PLLA groups. All photographs were taken at 4× magnification. * New bone.

**Figure 9:**
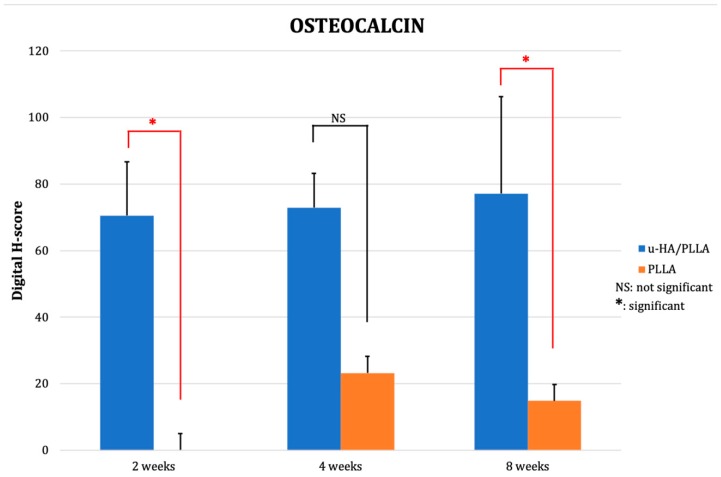
The mean digital H-scores based on immunohistochemical staining with anti-OCN antibody for the u-HA/PLLA and PLLA groups at weeks 2, 4, and 8. At week 2, there was limited new bone formation in the u-HA/PLLA group, whereas no new bone was observed in the PLLA group. The expression of OCN in the u-HA/PLLA group remained relatively stable over time and was significantly higher than in the PLLA group at week 8. * *p* < 0.05.

The digital H-score of OCN in the u-HA/PLLA group went up slightly from 70.56 at week 2 to 72.98 at week 4 and 77.20 at week 8. In the PLLA group, there was no new bone observed at week 2. From weeks 4 to 8 the digital H-score dropped from 23.23 to 14.84. At week 8, the discrepancy between the two groups was significant (*p* < 0.05) (Figure 8 and Figure 9).

## 4. Discussion

When it comes to osteosynthetic reconstructive materials, titanium has been the gold standard in maxillofacial reconstruction surgery since its introduction many years ago. With recent innovations in biotechnology, several bioresorbable alternatives have become commercially available. The results from using these innovative devices are comparable to those from using traditional titanium plates and screws [3,25,26,27,28,29]. Therefore, with the advantage of no removal requirement, biodegradable materials will soon replace titanium in the management of maxillofacial trauma or osteotomy, although they remain biomechanically weaker. However, the use of these resorbable devices remains limited due to some negative characteristics. PLLA belongs to the first generation of bioresorbable material used to manufacture bone osteosynthetic plates and screws; these have been utilized with some success in orthopedics and maxillofacial surgery [30,31]. Unfortunately, the application of PLLA is limited to non-complex fractures and low load-bearing areas. PLLA is weaker than titanium and causes some foreign-body reactions; moreover, it has a long resorption time [32]. More importantly, PLLA does not have any osteoconductive ability. An animal study conducted by Shikinami and colleagues in 2005 showed that a bone hole implanted with a PLLA rod remained unfilled at 5.5 years after implantation [33]. Compared to PLLA and other bioresorbable materials, u-HA/PLLA is notable for its mechanical strength, ease of use, bioactive properties and osteoconductive ability. This is the first animal study to confirm the feasible bioactivity and osteoconductivity of u-HA/PLLA in the maxillofacial region compared with PLLA and a sham control. Results obtained from investigating the effectiveness of using a u-HA/PLLA sheet to cover a mandibular critical-size defect may be applicable to the treatment of fractures and other maxillofacial bone defects in humans.

Our histomorphometric and histological assessments revealed that a significant amount of new bone formed at the defect area, especially at 8 weeks post-surgery in the u-HA/PLLA group. In some specimens, the new bone was observed to have direct contact with the parent bone and also with the u-HA/PLLA material on both sides, while the PLLA group only exhibited limited amounts of new bone growth at weeks 4 and 8 and the new bone was located only at the defect area. The results from the PLLA group were in agreement with those reported by Amano and colleagues [34]; i.e., the bone regeneration only occurred in the space created by the PLLA membrane, which could be explained by the guided bone regeneration (GBR) principle with the PLLA sheet acting as a barrier [34,35]. On the other hand, in the u-HA/PLLA group, the bone regeneration phenomenon may be due to a similar GBR mechanism [36]. Moroi et al. published a study in 2013 using either a u-HA/PLLA or titanium mesh as the GBR membrane to reconstruct a rabbit mandibular defect [37]. In that study, a semicircle defect was made at the inferior border of the mandible. Then, the defect was covered by either a u-HA/PLLA or titanium mesh to prevent soft tissue migration from all aspects. Their results revealed that new bone occupied nearly 90% of the space between the membrane and the host bone at week 4 in the u-HA/PLLA group as measured using histomorphometry. In our study, the mean new bone area achieved in the u-HA/PLLA group was only approximately 35% at week 4 and more than 60% at week 8. Although the comparison of results between our study and Moroi et al. [37] is not appropriate because of differences in study design, the amount of new bone formed in our study was still considerably less. However, in Moroi et al. [37], all aspects of the defect were protected from soft tissue contact, whereas in our study, the defect was a through-and-through perforation and the u-HA/PLLA sheets were only placed on the buccal side, hence it was not an ideal condition for GBR. In fact, for the PLLA-treated rats, which underwent the same surgical method to place the repair material, new bone was only present in specimens with an intact lingual periosteum, whereas in other PLLA specimens, the medial pterygoid muscle occupied the defect space and no new bone was observed. Therefore, the new bone in the u-HA/PLLA subjects in our study was unlikely to be formed via the GBR mechanism alone; instead, it was likely mainly formed by the intrinsic characteristics of the repair material. As demonstrated previously, the u-HA/PLLA material has been observed to have a layer of calcium phosphate crystals surrounding the material after immersion in simulated body fluid in vitro, because u-HA particles are exposed in the composite due to the machining process during manufacture [6]. This layer could be seen as early as 3 days after implantation, and it completely surrounded the material after 7 days. The calcium phosphate layer also gives u-HA/PLLA its osteoconductive features [38] and could explain why significant bone formation and bonding with both host bone stumps occurred in our study. With the material only covering the buccal side of the critical defect and an “open” lingual side, the bone regeneration capacity of the u-HA/PLLA sheet can still be considered significant. Furthermore, the new bone was deposited along almost the entire length of the u-HA/PLLA sheet on both sides at week 8, and was continuous with the parent bone. In other words, almost all of the critical size defect was filled by new bone. This phenomenon, which was also observed in a human biopsy with u-HA/PLLA screws [10,39], in addition to the abovementioned osteoconductive potential of u-HA/PLLA, showed that it may be applied effectively in the maxillofacial area where injuries that communicate with the external environment, such as maxillary sinus wall fractures, frontal sinus anterior table fractures, or orbital wall defect fractures, are frequently seen. Some clinical studies that researched the effectiveness of using u-HA/PLLA sheets to treat orbital floor fractures have reported favorable outcomes after a long follow-up time [14,40]. Further in vivo studies designed for these specific purposes should be considered in the future.

Runx2 is a transcription factor that is essential for the differentiation of osteoblasts [41]. In this study, Runx2 was highly expressed in the u-HA/PLLA group at week 2, which indicated that differentiated osteoblasts were present. The labeling index value then decreased as the newly formed bone matured. By contrast, the labeling index values of Runx2 were relatively stable in the PLLA group. This implied that the osteoblastic activity was more intense in the u-HA/PLLA group than in the PLLA group. Similar patterns of change in Runx2 labeling have been reported by other studies in which immunohistochemistry with anti-Runx2 protein was used for the assessment of bone substitute material [42,43,44]. As Runx2 has a role in osteoblast differentiation, the elevated expression of Runx2 at week 2 could be associated with subsequent new bone formation. Additionally, at week 2, the cells positive for Runx2 were located at the periphery of the defect as well as in the fibrous cells at the center of the defect. This may indicate that the new bone was derived from both the resting osteoblasts in the host bone and the mesenchymal stem cells that migrated to the defect. This theory is supported by the osteoconductive potential of the calcium phosphate layer on the u-HA/PLLA surface as well as our histopathological observations. Namely, new bone was observed to be continuous with parent bone in some specimens, whereas in other specimens, the new bone was formed without any connection in the center of the defect. Additionally, this finding also suggests that bone formation is promoted by partial contact with u-HA/PLLA material. A fracture model to test this theory should be carried out in the future. 

OCN is a non-collagenous bone matrix protein secreted by osteoblasts that binds to calcium ions and HA [45]. The digital H-score of the u-HA/PLLA group remained stable and relatively high, increasing only slightly from week 2 (70) to week 8 (77). In the PLLA group, OCN detected in new bone was only observed at week 4 (23.23) and the digital H-score declined to 14.84 by week 8. The difference in score between the two groups was obvious; however, it was only statistically significant at week 8. This could be a result of the small sample size included in this study. OCN expression in the u-HA/PLLA group indicated that the new bone formed in this group was mineralized and reached maturity earlier, and was more abundant, than in the PLLA group. This pattern was consistent with the findings of other studies that examined osteoconductive materials [42,46,47], in that the expression of OCN in the new bone of the study groups remained at a high level compared to that in the control groups. This finding was also consistent with the results of the histological and Runx2 evaluations.

There were a few limitations to this study. Firstly, the use of a rat model may prevent generalization of the results to humans, due to differences between the two species. However, based on the successful application of u-HA/PLLA in the maxillofacial region reported in numerous clinical investigations, the similar outcomes may be expected in human subjects. Human studies with sophisticated methodologies involving bone biopsy and computed tomography should be conducted to confirm our findings. Secondly, because of ethical concerns, the number of subjects was relatively low, resulting in a risk of bias. Thirdly, the follow-up duration was limited to only 8 weeks, so complete replacement of the u-HA/PLLA with bone could not be assessed, as performed by Shikinami and colleagues [33]. A long-term in vivo experiment should be conducted to assess resorbability and bony replacement in the maxillofacial region.

Despite these weaknesses, the results of our evaluations were all in agreement and showed that it was feasible to form new bone in a critical-size defect in a rat mandible with a u-HA/PLLA sheet. More newly regenerative bone was formed, and it matured much earlier than the new bone in PLLA subjects. This study confirmed the osteoconductivity of u-HA/PLLA used as a reconstructive device in the maxillofacial region. In addition to its superior strength, bone bonding, and radiopacity, the successful outcomes of using the u-HA/PLLA sheet in the rat mandible model suggests that it can be designed to be used effectively in not only human mandibles but also other human maxillofacial bones. In future studies, we would like to examine the mineral density and mechanical strength of bone formed with the aid of a u-HA/PLLA device.

## 5. Conclusions

The histological and immunohistochemical evaluations in this study have confirmed the bone regeneration capability of u-HA/PLLA material in the rat mandible model. These results may lead to the development of novel applications of u-HA/PLLA osteosynthetic reconstruction materials in the maxillofacial region and could shed light on the clinical feasibility and outcomes of using u-HA/PLLA in maxillofacial reconstructive surgery. Future studies examining the application of u-HA/PLLA in situations simulating specific clinical settings should be considered.
